# WALIS dashboard: An online tool to explore a global paleo sea-level database

**DOI:** 10.12688/openreseurope.16183.1

**Published:** 2023-07-14

**Authors:** Sebastián Garzón, Alessio Rovere

**Affiliations:** 1 Laboratory of Geo-information Science and Remote Sensing, Wageningen University & Research, Wageningen, Gelderland, 6708PB, The Netherlands; 2 Department of Environmental Sciences, Informatics and Statistics, Universita Ca' Foscari, Venice, 30172, Italy; 3 MARUM, Center for Marine Environmental Sciences, University of Bremen, Bremen, 28359, Germany

**Keywords:** Past sea level change, Geological database, Last Interglacial, Data visualisation, Sea-level changes, Paleoclimate

## Abstract

This paper describes the WALIS dashboard, an open-access interface to the World Atlas of Last Interglacial Shorelines (WALIS), which was developed and compiled thanks to funding from the European Research Council. WALIS is a database that includes thousands of samples (dated with different radiometric methods) and sea-level indicators formed during the Last Interglacial (125 ka). The WALIS dashboard was coded in R (shiny app) and is connected with a simplified version of the WALIS database. The app allows querying data by geographic extent or by filtering metadata. It then allows the user to download the queried data and perform simple reproducible data analysis. The WALIS dashboard can be used both online and offline.

## Plain language summary

Tide gauges and satellites provide reliable measurements of sea-level changes since the beginning of the 20th century. To estimate sea-level changes before this period, we rely on sea-level indicators, i.e., geological features that were formed in close connection with sea level in the past, such as fossil shallow-water coral reefs or cemented beach deposits. Similar to tide gauge and satellite data, data on sea-level indicators are collected and standardised in databases, which are then made available to the scientific community (and the public at large) for further analysis. In this work, we present an open-source application that allows exploring, analysing, and downloading sea-level indicators included in the World Atlas of Last Interglacial Shorelines (WALIS), a paleo sea-level database compiled thanks to funding from the European Research Council. The application aims to facilitate access to this information for researchers, students, and citizens by creating more interactive and intuitive ways to explore scientific information.

## Introduction

Geological indicators of past sea levels are fundamental to asses how ice sheets melted in the past and provide fundamental benchmarks to define possible scenarios of ice sheets melting in a warmer future climate ^1^. To be used as a sea-level indicator (or "sea level index point") a geological feature must be assigned an elevation and geographic location, an age via radiometric methods or stratigraphic correlation, and must have a quantifiable relationship with a former sea level ^2^. Knowing these three parameters allows for reconstructing the elevation of the relative sea-level at a point in time in the past. After correction for land motions (e.g., tectonics, or glacial isostatic adjustment ^3^), it is possible to reconstruct the global mean sea level at the time the sea-level indicator was formed.

The advent of data digitalisation has provided paleo sea-level researchers with new opportunities to discover and access studies from different research groups. Among the tools facilitating the exchange of information, scientific articles and open-access repositories have opened the possibility to download and analyse sea-level data to anyone with internet access. However, access to new studies and data comes with additional challenges for researchers. A widespread challenge is that the information related to sea-level indicators is communicated in multiple ways (e.g., graphs, tables, in-text explanations, supplementary information) that require readers to navigate between different styles and conventions. Sea level researchers often face additional challenges as a correct interpretation of data requires an understanding of several measurement and dating techniques, and requires in-depth knowledge of how the original information (e.g. stratigraphic context of geological sea-level index points) was interpreted by the authors. Recent standardisation efforts among the sea-level research community have resulted in standardised formats designed to store and share sea-level indicators in a way that allows different researchers to understand the origin and details of their measurements and reproduce the process to extract their components ^4, 5^. *The World Atlas of Last Interglacial Shorelines* (WALIS) is a standardised database that includes data from thousands of studies since the early 1900s. The focus is on sea-level indicators formed during marine isotope stage 5 (MIS 5) ^6^. The database includes 4545 sea-level proxies and 4110 dated samples standardised from 2130 references compiled by multiple research groups within a special issue of the Earth System Science Data (ESSD) journal. The structure of the database consists of multiple tables with one-to-one or many-to-many relationships that are openly available in Zenodo ^7^ in different export formats (e.g., CSV and geoJSON). In this work, we present an online dashboard that allows exploring WALIS data without having to download the database and with no coding knowledge required.

## WALIS Dashboard

**Figure 1.**
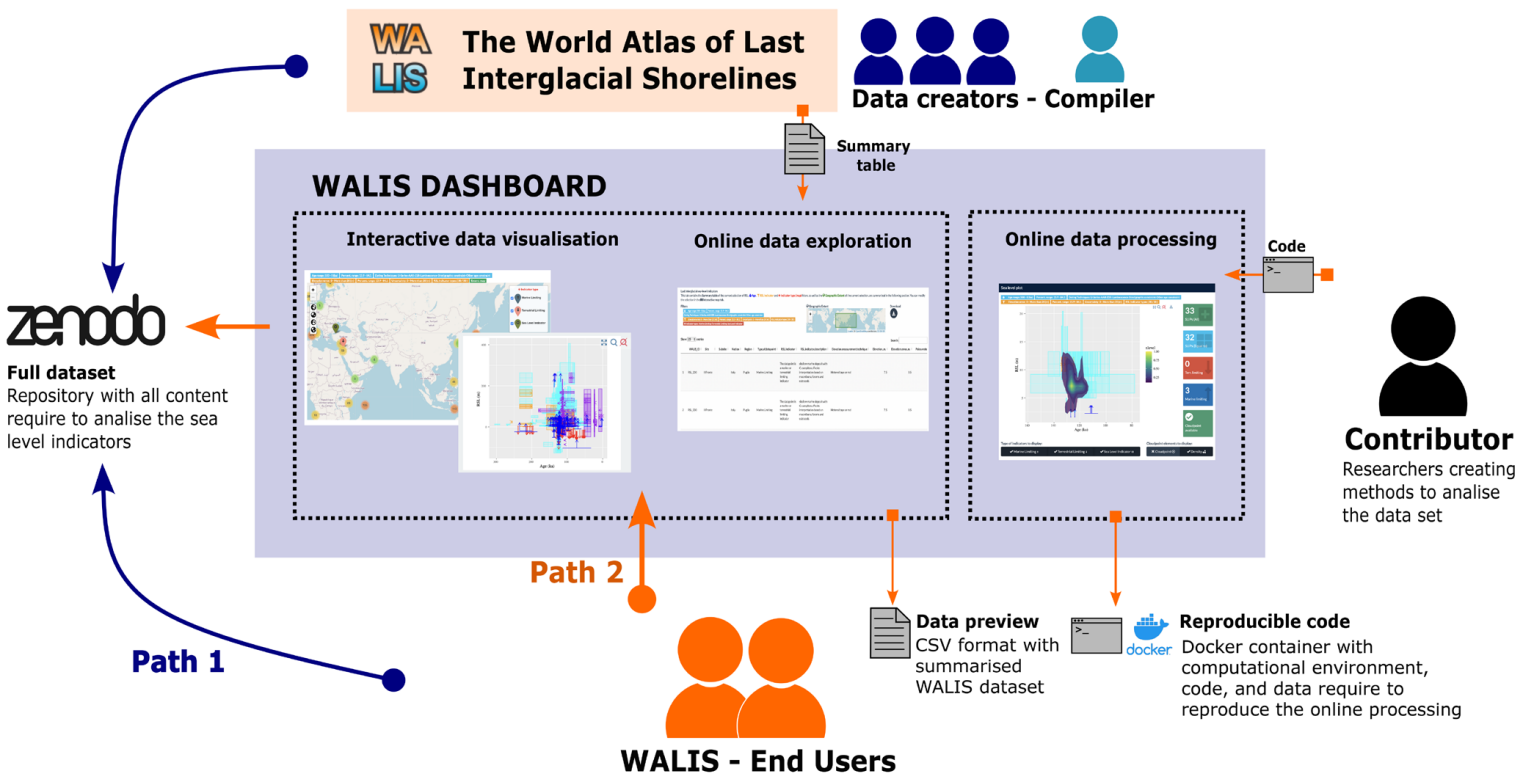
WALIS Dashboard diagram.

The main purpose of the WALIS Dashboard is to provide an alternative entry point for users to explore the WALIS database information (WALIS is licensed under CC-BY 4 and our use falls within the extent of this license). Figure 1 shows how the dashboard serves as a connection point between data creators, data compilers, external contributors, and end-users around the WALIS database. Traditional access for end-users of WALIS would require them to directly download the full dataset from Zenodo. Given the complexity required to standardise sea level indicators, these tasks would require users to familiarise themselves with the dataset structure before exploring the content. Similarly, end-users would need to develop their own data visualisation methods to find relevant information in the dataset. Given the challenges that might discourage or make dataset exploring difficult, the WALIS dashboard provides an alternative path for a user to easily get an idea of the content of the dataset provided by the data creators and data compiler. Additionally, the dashboard integrates external contributions from researchers to explore the content of WALIS within a selected region of interest. All relevant documentation are provided in *Underlying data* ^8^ and *Software availability* ^9^.

### Implementation

The WALIS Dashboard is an interactive application developed using open-source R packages. The application is built using the R-Shiny package (RRID:SCR_001626) ^10^ that allows integration of data and analysis in an interactive web platform. The application includes individual data visualisations often used in the literature to provide context about sea-level indicators such as maps, sea-level plots, and tables. Additionally, the application allows users to apply a Monte Carlo method proposed by 11 to merge and summarise multiple sea-level indicators using the WALIS database structure.

To display this information the application is divided into three tabs: (1) Interactive map, (2) Summary table, and (3) Merge SLIP. These are briefly described below.

### Interactive map

**Figure 2.**
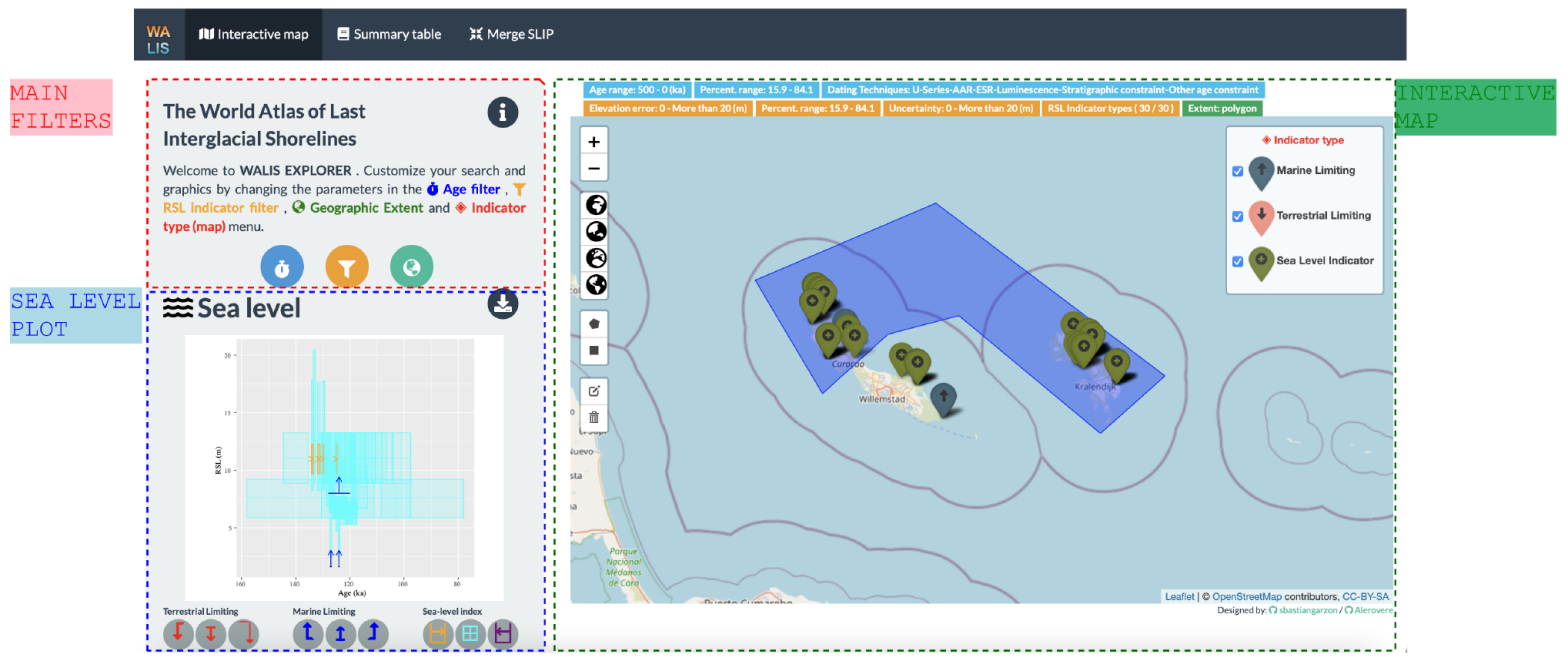
Interactive map tab showing a selection of sea-level indicators in Curaçao and Bonaire.

The interactive map tab integrates filtering options of the data set, a map visualisation, and a sea-level plot ( Figure 2). In our design, we maintain important elements of the structure of the data set regarding both measurement and interpretation of the data as proposed by 4.

**Figure 3.**
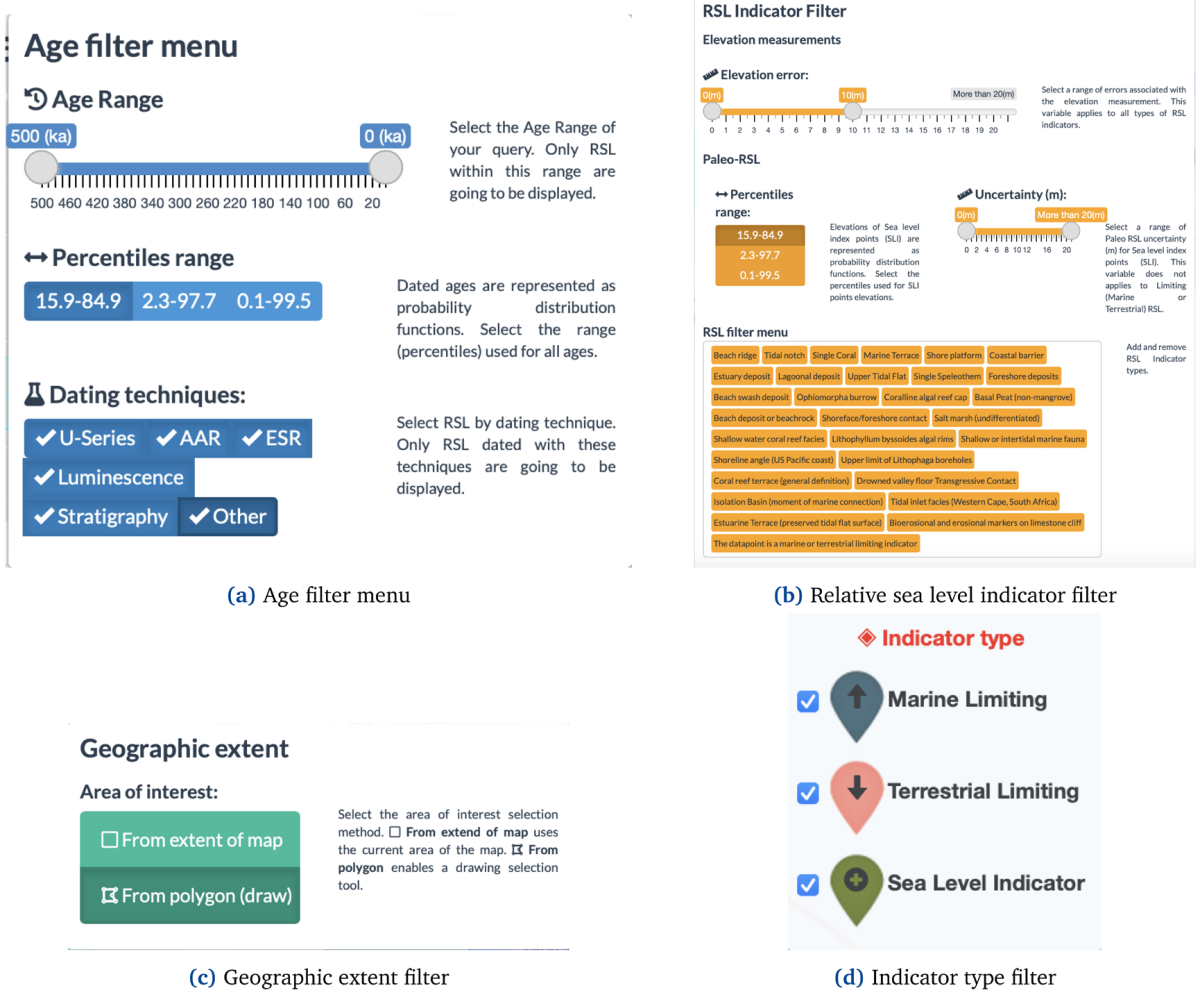
WALIS dashboard filters.

The filtering option ( Figure 3) allows selecting by type of indicator (i.e., Sea-level index point or limiting data) and allows culling the selection by age, relative sea-level (RSL), and location using other panels and elements as input. The "Age filter menu" ( Figure 3a) allows users to trim the data by timeframe of interest, age uncertainty, and dating techniques. The "RSL Indicator filter" ( Figure 3b) allows instead to selection of sea-level indicators by type, elevation error, paleo-RSL percentiles, and Uncertainty. The WALIS Dashboard also allows resampling the database by Geographic extent ( Figure 3c), showing information only within a particular area of interest. Via this interface, the user can either select data in the current map area or draw an area of interest.

Based on the filters applied to the data, the map visualisation displays the sea-level indicators with pop-ups with additional information. These include data and metadata on elevation measurements and dating, interpretation, and literature references.

The sea-level plot is the main element of data visualisation in the "Interactive map" tab. Based on the filters applied to the data, a graph is updated in real-time, displaying sea-level indicators in a sea-level vs time plot. In the graph, we implemented a symbology for the nine types of sea-level indicators and associated ages resulting from the WALIS structure. These new symbols represent particular cases of the three main types of indicators: Sea-level index points (SLIP), terrestrial limiting, and marine limiting. The platform includes details for all nine types of indicators clarifying their meaning for the temporal and relative-sea level dimensions.

### Summary table

**Figure 4.**
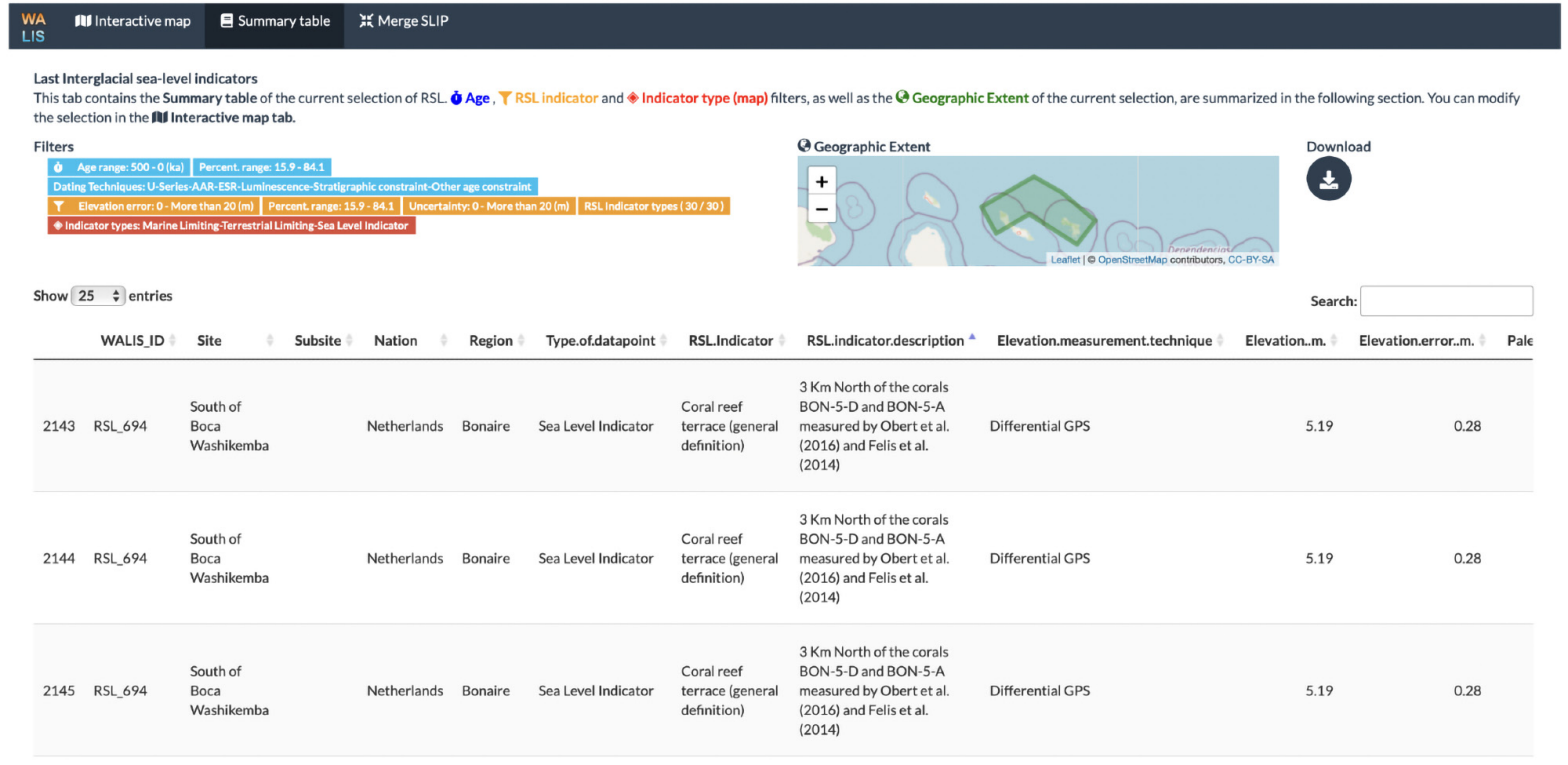
Summary table tab showing a selection of sea-level indicators in Curaçao and Bonaire.

The "Summary table" tab includes the information on the data as filtered in the "Interactive map" tab. As a visual guide, this tab displays the current filters and a map with the area of interest. The table includes additional information on the sea-level indicators and allows users to explore the content before using the export options available in the application. Additionally, users can download the summary table from this tab in CSV format. Figure 4 shows the summary table tab showing a selection of sea-level indicators in Curaçao and Bonaire.

### Merge SLIP

**Figure 5.**
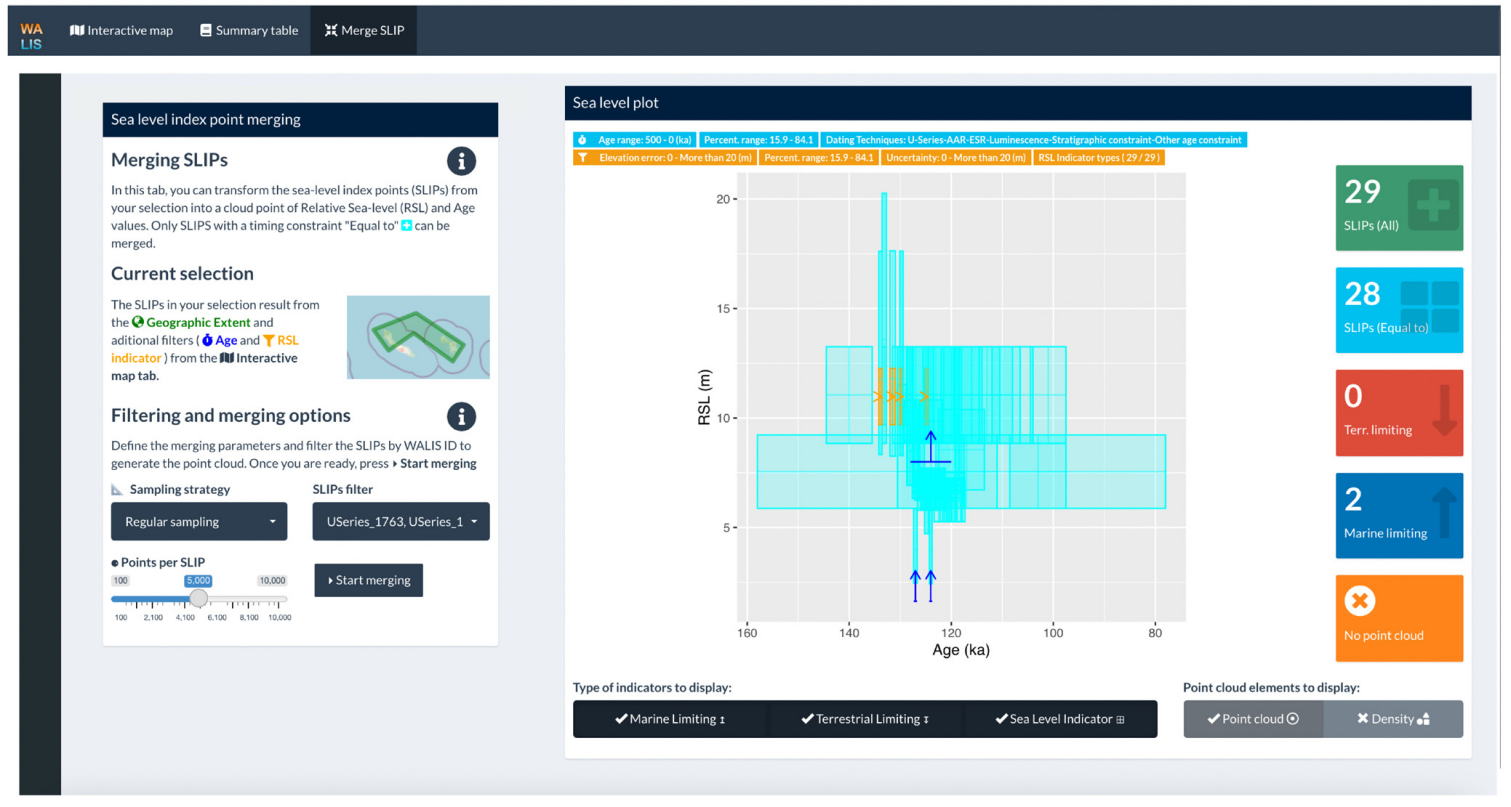
Merge SLIPs (Sea Level Index Points) tabs showing data selection in Curaçao and Bonaire.

The "Merge SLIP" tab allows the user to create a point cloud of Relative Sea-level (RSL) and Age values with the sea-level index points that were selected in the "Interactive map" tab ( Figure 5). The merging method follows the methodology proposed by 11 to combine the different probability distributions of Age and RSL values of each sea-level index point (SLIP) into a single point cloud. Before merging the data, the user can further filter the dataset, modify the sampling strategy, and determine the number of points to be sampled.

After the sea level index points are merged, the user can explore the results in a sea level vs age plot. The results can then be exported using two different strategies: Point cloud download or Docker container. The first option exports the resulting point cloud into a CSV file accompanied by a geoJSON file with information about the filters used to extract the selection of data. The second option creates a docker image accompanied by the required data and code to fully reproduce the results of the data merging. Therefore results obtained remotely in the Dashboard are fully reproducible on a local machine.

### Operation

***System requirements***

The WALIS dashboard can be accessed both online and offline. The online version is available as a shiny app here (Last access July 3, 2023). The target for the online app is users that need to explore the WALIS data set (e.g., sea-level researchers, students). Users do not need to install any R package or manipulate any code. Access to the online version only requires a stable internet connection. The dashboard can be accessed in a local build after downloading the source code available on our GitHub repository or in the Zenodo repository ^9^. The software was developed using R (RRID:SCR_001905) version 4.1.0 ^12^. The target for the offline version is researchers that want to contribute to expanding the capabilities of the dashboard. Contributions are welcome as new issues or pull requests in the main GitHub repository.

***Local shiny application***

Users can deploy a local implementation of the shiny application using the {renv} package. This option provides the required files and R packages information. An R installation is a prerequisite for this option.

1. Download the WALIS dashboard from GitHub - see *Software availability* ^9^

2. Open R and install the *renv* package.
install.package("renv")

3. Open the R Project file (WALIS_Visualization.Rproj). This file should start the process to restore the dependencies of the project using the Lockfile (renv.lock). You can manually restore the dependencies using the function restore() from the R package *renv*
library("renv")
restore(renv.lock)

4. Open the app.R file.

5. Run the App using the runApp() function from the Shiny R package.
library("shiny")
runApp(your_path/to/app.R)

***Docker image***

A Docker image to run the application is available as part of the application. This docker image allows the application to be fully reproducible as the instructions and computational requirements (e.g., operating system, R packages) to deploy the application inside a software container are automated. The only prerequisite is to have Docker (see 13) installed and running on the local machine. This option is mostly available to guarantee the reproducibility of the application in the long term.

***Download and start-up instructions - Docker***

1. Download the WALIS dashboard from GitHub - see *Software availability* ^9^

2. Open Docker to run in the background

3. Open a terminal and access the folder with the application
cd WALIS_Visualization

4. Create a Docker container using the Dockerfile image. This process could take hours the first time as it requires setting up all computational requirements.
docker build -t ’walis-shiny’.

5. Run the Docker container
docker run -p 3838:3838 ’walis-shiny’

6. Open the application in a web browser at *http://localhost:3838*

## Use cases

### Use case #1: Discovering sea level indicators

This use case illustrates the most common use of the tool to discover sea-level indicators in an area during a specific time period. In this case, we illustrate how a researcher can find sea-level indicators under multiple restrictions:

1. Only Sea Level Index Points (SLIPs)

2. SLIPs around the Last Interglacial (130.000 to 115.000 ka).

3. SLIPs dated using Uranium-Thorium (U-Series) dating.

4. SLIPs located in Bonaire and the Northern Part of Curaçao

5. SLIPs with elevation measurement errors below 10 meters.

**Figure 6.**
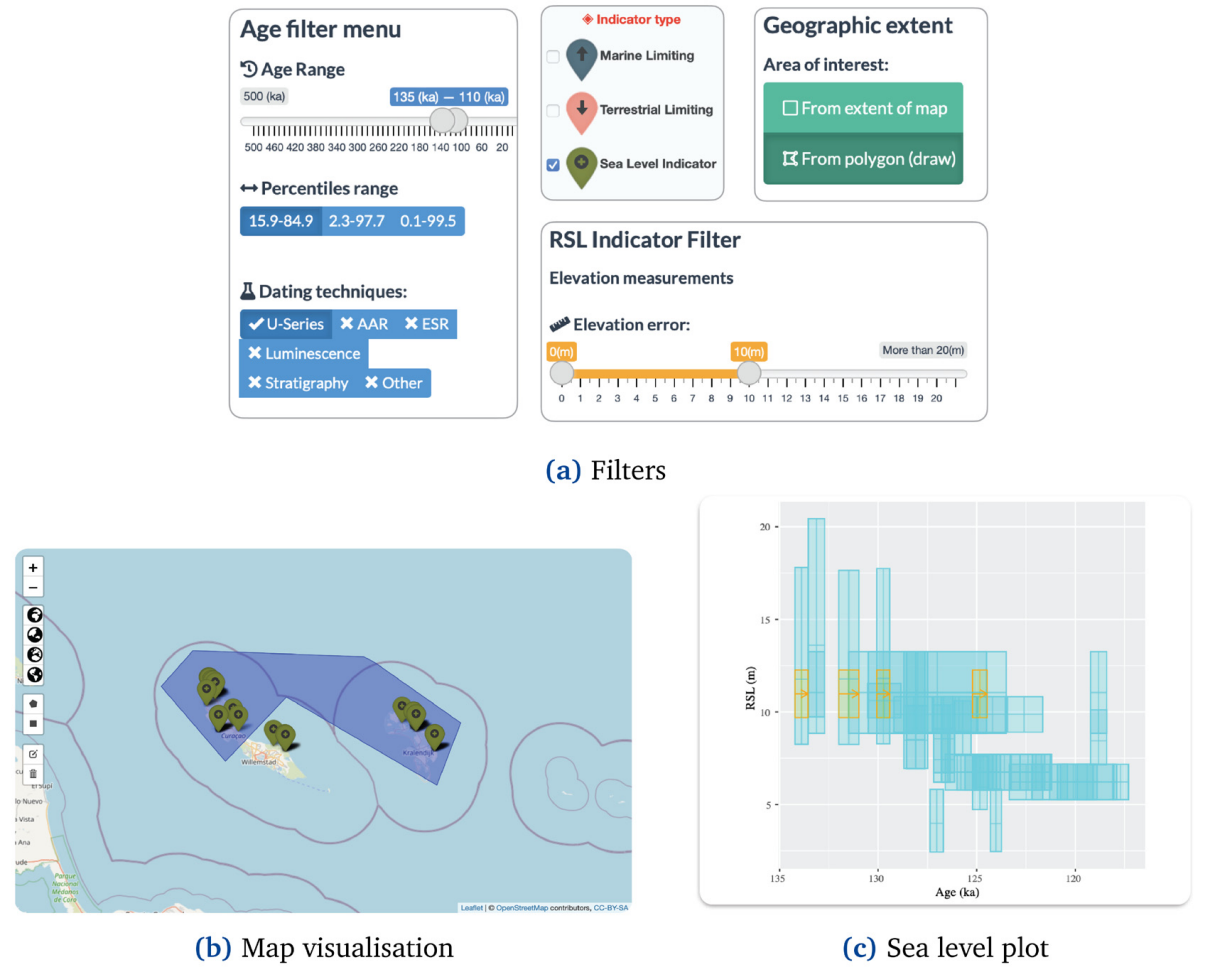
Use case 1: Discovering sea level indicators.

Figure 6a shows the filter configuration required to select sea level indicators under the conditions. First, the user can exclude Marine Limiting and Terrestrial Limiting indicators using the "Indicator type" menu located on the Map. Second, in the Age filter menu, the user can select the desired period around the Last Interglacial (e.g., 135 to 110 ka to include additional data) by modifying the Age range slider. In this menu, the user can exclude other dating techniques (e.g., Amino acid racemisation -AAR- and Luminescence) by clicking on them. Third, in the RSL Indicator filter user can modify the elevation error slider to only include the range between 0 and 10 meters. Finally, the user should enable the "From polygon (draw)" option in the Geographic extent filter menu. After enabling this option, the user can draw a polygon that excludes the southern part of Curaçao ( Figure 6b).

Multiple observations can be made after data selection. For example, there are 21 sea-level indicators ( *e.g., RSL_3559* and *RSL_3553*) with a total of 48 individual constraints in the area. Taking all individual constraints into account, the sea level plot shows that the relative sea level during the Last Interglacial in this area fluctuated between 2.5 and 20 meters. A rapid scan using the Summary Table tab shows that relevant literature for the search includes, among other five articles, 14 and 15. A summary of these newly discovered sea-level indicators under a series of specific conditions can be downloaded for further inspection using the download menu available both in the Interactive Map and Summary Table tabs. The WALIS_ID field from all sea-level indicators found in the search will enable users after downloading the full WALIS database to easily identify sea levels in this area.

### Use case #2: Estimating relative sea level

Using the selection of sea-level indicators from the previous use case we can estimate the relative sea-level in this area. As relative sea-level indicators can have more than one constraint, analysing the sea level plot from Use case #1 might not be sufficient to estimate the sea level in the area. For example, in our selection, there are sea-level indicators with five individual constraints. In the sea-level plot, this would result in five more relative sea level (RSL) points (i.e., symbols in the plot) than a sea-level indicator with only one constraint. Therefore, for estimating RSL our strategy is to merge individual constraints and compare only the final sea-level indicators using the methodology proposed by 11. This methodology summarises sea level indicators by creating a point cloud based on individual constraints. All sea level indicators have the same final number of points no matter the initial number of constraints per indicator.

**Figure 7.**
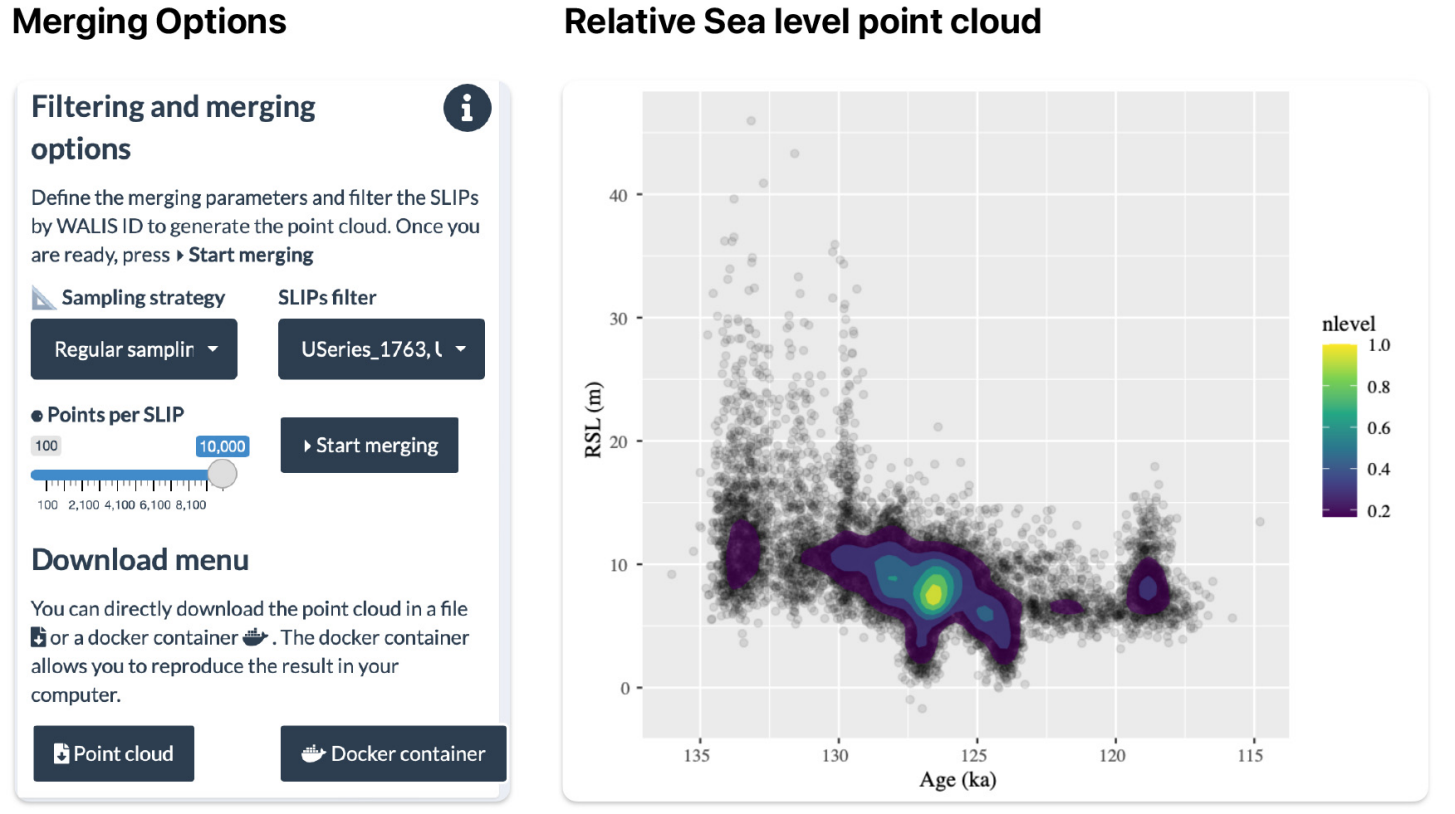
Use case 2: Merging Sea level indicators.

Users can create a point cloud of relative sea level values using the Merging SLIP tab. Figure 7 shows the filtering and merging options required to estimate the relative sea level for this area based on the selection. First, the user selects a sampling strategy among two possible options proposed by 11: Regular sampling and Peak sampling. Generally, Regular sampling is the default merging strategy while Peak sampling is only relevant for sea level indicators with uniform temporal constraints. For example, sea level indicators in which the age calculation comes from a Uniform distribution from a Marine Isotope Stage (MIS) assignment. Second, the user can use the SLIPs filter to remove individual sea-level indicators by code if required. Finally, the user can select the number of points per sea-level indicator. The upper limit of 10.000 points per indicator is set to not overload the application with calculations.

After processing, the user can see the relative sea level point cloud ( Figure 7) and include a density calculation. Based on the selected data, we can estimate that around 130 and 125 ka relative sea level in Bonaire and the Northern Part of Curaçao was around 12 and 5 m with a decreasing tendency. Both the sea level plot and the point cloud can be downloaded using the Download menu.

### Use case #3: Reproducing the results

The analysis performed in Use Case #2 can be extended if combined with other sources of sea level information. For example, RSL point clouds can be combined with Global Isostatic Adjustment (GIA) models from the area as described by 11. For these additional analyses, it is important to keep track of the origin of the point clouds and how this source of sea level information was obtained.

**Figure 8.**
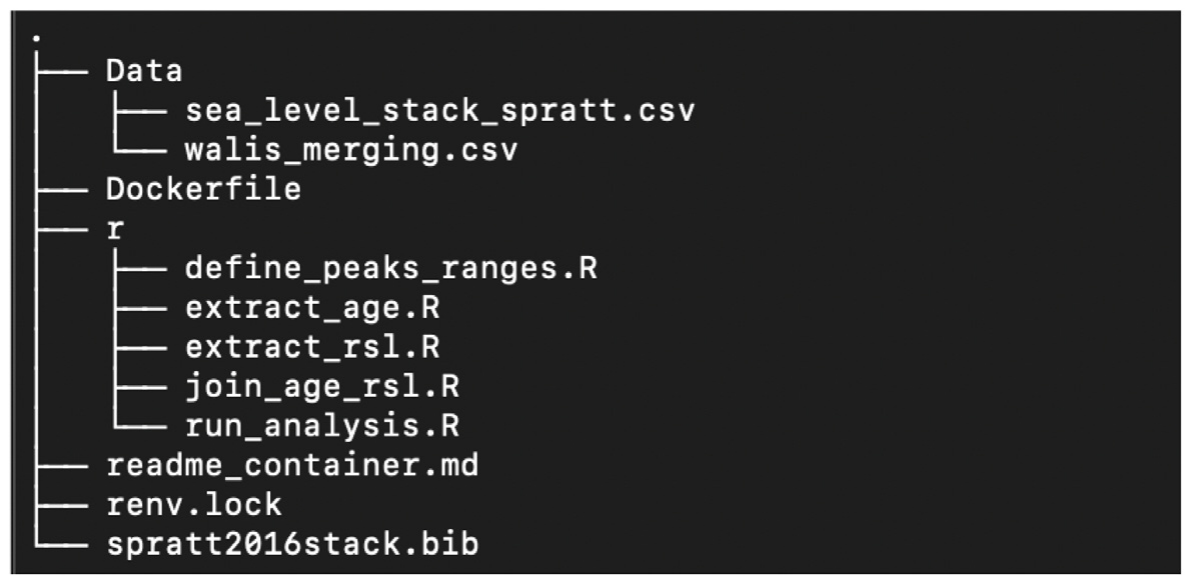
Use case 3: Tree structure docker container folder.

A folder with all required information is available to guarantee that any analysis performed in the dashboard can be reproduced outside the application ( Figure 8). This folder includes a *Data* subfolder with all SLIP in the area in CSV format, an *R* subfolder with all scripts required to perform an analysis, a *renv.lock* file to keep track of all required R-packages, a *Dockerfile* to generate the container, and a *readme_container.md* file with detailed instructions on how to reproduce the analysis. To reproduce the results of the analysis and generate the same point cloud as in the dashboard, users need to follow these instructions:

1. Download the Docker container in the Download menu

2. Open Docker to run in the background

3. Open a terminal and access the folder
cd WALIS_docker

4. Create a Docker container using the Dockerfile image. This process could take a couple of minutes depending on the characteristics of the local machine.
ddocker build -f Dockerfile_container -t ’walis-ok’.

5. Run the Docker container
docker run -p 80:80 walis-ok

6. To extract the result from the container (CSV file with point clouds) users need to transfer the file from the container to their local machine. For this, users need to identify the CONTAINER_ID.
docker ps -a

7. Using the 10-character {CONTAINER ID} string users can now transfer the file to their local machines.
docker cp {CONTAINER ID}:/merging/pointcloud_output.csv.

8. The pointcloud_output.csv file will be available now in the WALIS_docker folder

In addition to reproducing the analysis performed using the dashboard, the content of this folder provides the user with all the required information to perform their own individual analysis.

## Conclusions

In this article, we present an open-source online dashboard developed using R packages to explore the WALIS database. This dashboard provides an example of how open-source tools can be used to simplify access to database information for research teams with limited software development capabilities. The interactive application consists of three tabs that summarise the database information for researchers to provide a user-friendly point of connection to the information. The application includes basic data processing methods that provide meaningful observations for researchers to start analysing the content of the database. Given the application design, end-users of the application should be able to easily explore the WALIS database before engaging in more complex and time-consuming tasks to understand the database structure. To promote further developments and guarantee the long-term and offline maintenance of the application, the software includes reproducibility strategies such as software containers and dependency management strategies. Similarly, the application is licensed under an MIT permissive free software license to promote researcher teams to implement similar interactive visualisation approaches for other databases.

## Ethics and consent

Ethical approval and consent were not required.

## Data Availability

Zenodo: WALIS - The World Atlas of Last Interglacial Shorelines (Ver 1.0 final). https://doi.org/10.5281/zenodo.7348242 ^8^. This project contains the following underlying data: Atlas_Versions/Ver_1/Ver_1_0_post_review/Output/DB_Structure/Summary_full.csv (CSV file containing the WALIS summary table). Data are available under the terms of the Creative Commons Attribution 4.0 International Public License (CC BY 4.0).

## Software availability

Software available from: https://warmcoasts.shinyapps.io/WALIS_Visualization/

Source code available from: https://github.com/Alerovere/WALIS_visualization

Archived source code at time of publication: https://doi.org/10.5281/zenodo.8013088 ^9^

Licence: MIT
