# WALIS dashboard: An online tool to explore a global paleo sea-level database

**DOI:** 10.12688/openreseurope.16183.2

**Published:** 2024-03-01

**Authors:** Sebastián Garzón, Alessio Rovere

**Affiliations:** 1 Department of Physical Geography, Faculty of Geosciences, Utrecht University, Utrecht, The Netherlands; 2 Department of Environmental Sciences, Informatics and Statistics, Universita Ca' Foscari, Venice, 30172, Italy; 3 MARUM, Center for Marine Environmental Sciences, University of Bremen, Bremen, 28359, Germany

**Keywords:** Past sea level change, Geological database, Last Interglacial, Data visualisation, Sea-level changes, Paleoclimate

## Abstract

In this paper, we present WALIS Dashboard, an open-access interface to the World Atlas of Last Interglacial Shorelines (WALIS), which was developed and compiled thanks to funding from the European Research Council. WALIS is a database that includes thousands of samples (dated with different radiometric methods) and sea-level indicators formed during the Last Interglacial (~80 to 130 ka). The WALIS Dashboard was coded in R (shiny app), and allows querying a simplified version of WALIS by either geographic extent or by attributes. The user can then download the queried data and perform simple and reproducible data analysis. The WALIS Dashboard can be used both online and offline.

## Plain language summary

Tide gauges and satellites provide reliable measurements of sea-level changes since the beginning of the 20th century. To estimate sea-level changes before this period, we rely on sea-level indicators, i.e., geological features that were formed in close connection with sea level in the past, such as fossil shallow-water coral reefs or cemented beach deposits. Similar to tide gauge and satellite data, data on sea-level indicators are collected and standardised in databases, which are then made available to the scientific community (and the public at large) for further analysis. In this work, we present an open-source application that allows exploring, analysing, and downloading sea-level indicators included in the World Atlas of Last Interglacial Shorelines (WALIS), a paleo sea-level database compiled thanks to funding from the European Research Council. The application aims to facilitate access to this information for researchers, students, and citizens by creating more interactive and intuitive ways to explore the scientific information contained in WALIS.

## Introduction

Geological indicators of past sea levels are fundamental to assessing how ice sheets melted in the past and provide fundamental benchmarks to define possible scenarios of ice sheets melting in a warmer future climate ^1^. To be used as a sea level index point (SLIP, also called “relative sea-level indicator“), a geological feature must be assigned an elevation and geographic location, an age via radiometric dating or chronostratigraphic correlation, and must have a quantifiable relationship with a former sea level, called indicative meaning ^2^. Knowing these three parameters, it is possible to reconstruct the relative sea level (RSL) at a point in time in the past. In turn, RSL corresponds to the sum of global mean sea level and post-depositional land motions caused by different processes (e.g., tectonics, glacial isostatic adjustment, sediment compaction ^3^).

The advent of data digitalisation has provided paleo sea-level researchers with new opportunities to discover and access studies from different research groups. Among the tools facilitating the exchange of information, scientific articles and open-access repositories have opened the possibility to download, analyse and in some case visualize ^4^ sea-level data to anyone with internet access. However, access to new studies and data comes with additional challenges. A widespread issue is that the information related to sea-level indicators is communicated in multiple ways (e.g., graphs, tables, in-text explanations, supplementary information) that require readers to navigate among different styles and conventions. Sea level researchers often face additional challenges as a correct interpretation of data requires an understanding of several measurement and dating techniques, and requires in-depth knowledge of how the original information (e.g., the stratigraphic context of geological sea-level index points) was interpreted by the authors. Recent efforts among the sea-level research community have resulted in standardised formats designed to store and share information on sea-level indicators in a way that allows different researchers to understand the origin and details of their measurements and reproduce the process to extract their components ^5, 6^.

The World Atlas of Last Interglacial Shorelines (WALIS) is a standardised database that includes data from thousands of studies published since the early 1900s. The focus is on sea-level indicators formed during the Last Interglacial (~80 to 130 ka), although the database includes some older data points ^7^. The database includes 4545 sea-level proxies and 4110 dated samples standardised from 2130 references compiled by multiple research groups within a special issue of the Earth System Science Data journal. The structure of the database consists of multiple tables, that are openly available in Zenodo ^8^ in different export formats (e.g., CSV and geoJSON). In this work, we present a dashboard that allows exploring WALIS data, and can be used both offline and online. The basic use of the dashboard does not require downloading the database or extensive coding knowledge.

## The WALIS Dashboard

The main purpose of the WALIS Dashboard is to provide an alternative entry point for end users to explore the information included in WALIS. We note that our use of WALIS falls within the license under which the database is shared (CC-BY 4.0). The WALIS Dashboard was originally coded in the framework of the master thesis of Sebastián Garzón ^9^. The first version (ver. 1.0) of the WALIS Dashboard was released in 2021 ^10^. The second version (ver. 2.0) was released at the end of October 2022 ^11^ and is briefly outlined within the WALIS database description paper ^7^. For the peer-review of this manuscript, we prepared version 3.0, which includes bug fixes and improvements to the interface ^12^. Post-review, we released version 3.1 ^13^ , which considers the reviewer’s comments and proposes further improvements to the interface.

### The WALIS Database

The WALIS Dashboard is based on a simplified, single-table version, of the WALIS database (called Summary Table), which is included (together with the code used to generate it) in the WALIS database repository (see *Underlying data*). This table is created from the “Summary sheet” included in WALIS, calculating percentiles of the probability distribution for paleo RSL and age (0.1,2.3,15.9,50,84.1,97.7 and 99.5 percentiles). The RSL percentiles for each SLIP are calculated by applying, to each index point, the following procedure:

1. If the SLIP is a "Single Coral", the percentiles are obtained from a gamma function interpolated considering the upper limit of living range inserted in the database as, respectively, the 2.3 and 97.7 percentiles of the distribution.
2. If the RSL Indicator is a "Sea Level Indicator" or "Single Speleothem": the percentiles on paleo RSL are calculated from the Gaussian distribution represented by the field "Paleo RSL (m)" and its associated uncertainty (1-sigma).
3. If the RSL Indicator is a "Terrestrial Limiting" or "Marine Limiting", the RSL percentiles are not calculated.

For which concerns age percentiles, for SLIPs with radiometric ages they are calculated from the Gaussian distribution of the mean age and its associated 2-sigma uncertainty. In case the age of the SLIP is present in WALIS only as a time period (e.g., the SLIP is attributed to Marine Isotopic Stage 5), the age range is treated as a uniform distribution, with upper and lower age limits matching those indicated by Lisiecky and Raymo (2005) ^14^ and Spratt and Lisiecky (2016) ^15^, that are already coded into WALIS (e.g., a MIS 5 age assignment corresponds to an age range between 71 and 130 ka).

### Rationale and software description

WALIS is a complex database framework, as it includes several tables connected by several SQL relations (see Rovere *et al.*, 2023 ^7^ for a detailed description of the database). Traditional access to WALIS for end-users would require them to directly download the full dataset from Zenodo or connect to the SQL server that hosts the database. Given the complexity required to standardize SLIPs, these tasks would require end users to familiarize themselves with the dataset structure and with either Python or SQL to explore, visualize, or perform data analysis on the database. While the WALIS distribution ^8^ includes Python scripts to perform simple queries on the data, end-users would still need a good proficiency level in Python to use and modify these scripts to their needs. Given the challenges described above, we created the WALIS Dashboard to allow for a quick exploration, query, and analysis of the SLIPs included in the database. The WALIS Dashboard software architecture is described hereafter and the software is available for open access (see *Software availability*).

The WALIS Dashboard is an interactive application developed using open-source R packages. The software was developed using R (RRID:SCR_001905) version 4.1.0 ^16^. The application is built using the R-Shiny package (RRID:SCR_001626) ^17^ that allows the integration of data visualization and analysis in an interactive web platform. The application includes individual data visualisations often used in the literature to provide context on SLIPs, such as maps, sea-level plots, and tables. Additionally, the application provides the end users an interface to apply a Monte Carlo method ^9^ to merge and summarise multiple SLIPs within the same geographic context.

The WALIS dashboard can be accessed both online and offline. The online version is available as a freely-hosted shiny app here (Last access January 30 ^th^, 2024). We coded the online application targeting end-users who want to explore the WALIS data set without installing any R package or manipulating code. Access to the online version only requires a stable internet connection. The dashboard can be also accessed in a local build after downloading the source code available on our GitHub repository or in the Zenodo repository ^12^. The target for the offline version is researchers who want to contribute to expanding the capabilities of the dashboard, or who need to run more computationally intense data analyses. Contributions are welcome as new issues or pull requests in the main GitHub repository.

## Main features

**Figure 1.**
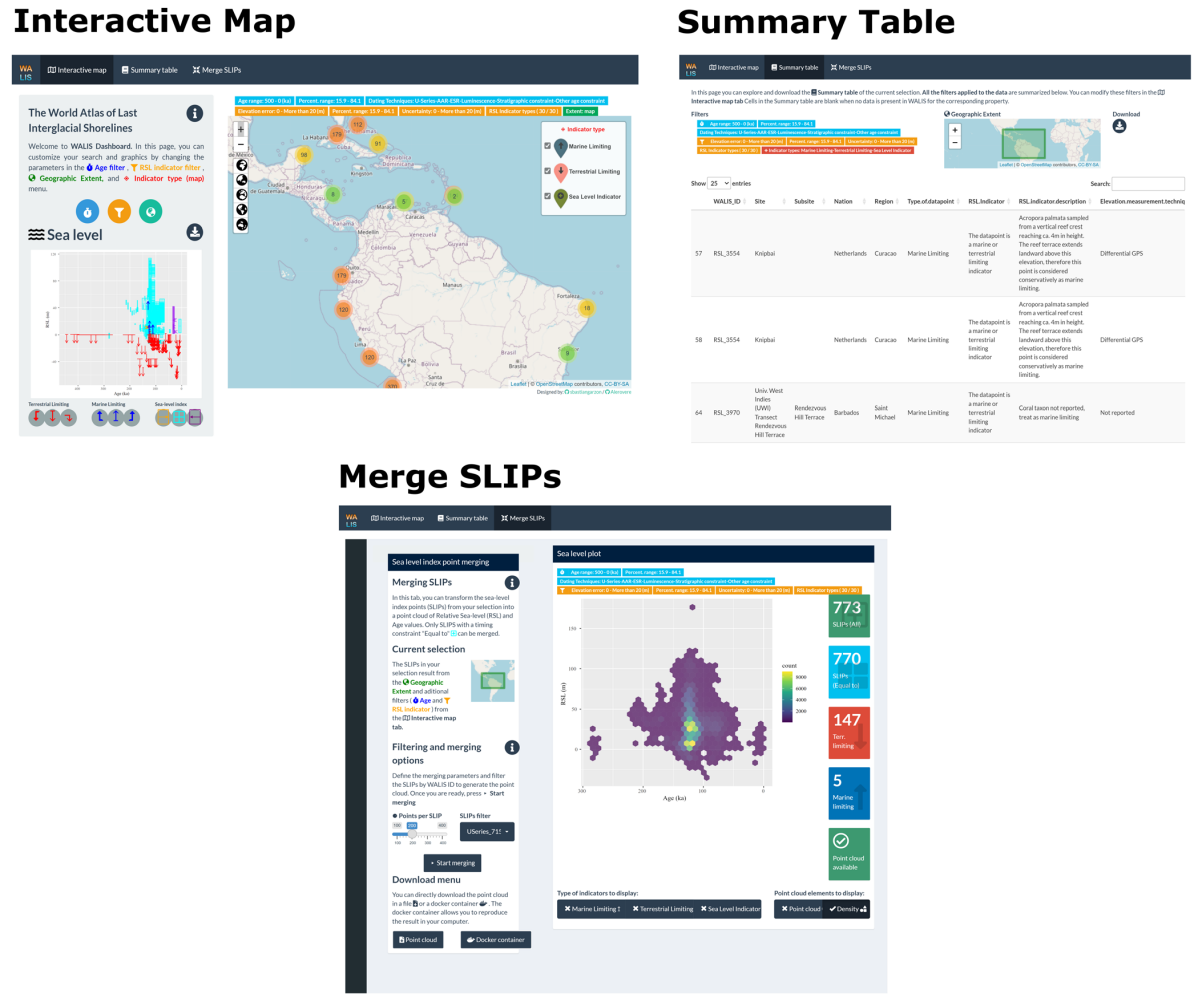
WALIS Dashboard interactive tabs.

The application is divided into three tabs: " *Interactive map*", " *Summary table*", and " *Merge SLIPs*" ( Figure 1). These are described hereafter one by one, but we remark that the operations (e.g., filtering) done on the data in one tab are propagated to the other ones.

### Interactive map

The starting page of the WALIS Dashboard is divided into three main parts: a filtering application, a map, and a sea-level plot. The filtering application allows querying the data through age properties (age range with user-specified percentile bounds and dating technique, SLIP properties (elevation error, RSL uncertainty and associated percentiles, and type of RSL indicator), and geographic extent (either gathered from the map display or via a polygon drawn by the user). The map shows the location of the SLIPs included in WALIS. Clicking on one point in the map, a popup menu appears with general information about the selected record. As a default, when panning and zooming onto the map the sea-level plot is updated. The sea-level plot shows RSL on the Y-axis and Age (ka) on the X-Axis and is the main element of data visualisation in the "Interactive map" tab. Based on the filters applied to the data, the graph is updated in real time. In the graph, we implemented a symbology for the nine types of sea-level indicators and associated ages allowed by the WALIS database ^7^. The sea-level plot can be downloaded in PNG format.

### Summary table

The second tab in the WALIS dashboard is called " *Summary table*" and includes the data as filtered in the " *Interactive map*" tab. As a visual guide, on the top of the page, two insets show the current filters active on the data and a map that defines the area of interest. The table displayed on this page includes all the information available from the simplified version of WALIS described in the previous sections. In the upper right corner of this page, a button allows downloading the data in CSV format.

### Merge SLIPs

The " *Merge SLIP*" tab allows the end-user to create a point cloud or density plot that represents a probability distribution of RSL vs age, using the SLIPs that were selected in the " *Interactive map*" tab. In this tab, the end-user can combine the different probability distributions of Age and RSL values of each sea-level index point (SLIP) into a single point cloud. Before merging the data, the user can further filter the dataset (by excluding selected index points) and determine the number of points to be sampled within each SLIP.

**Figure 2.**
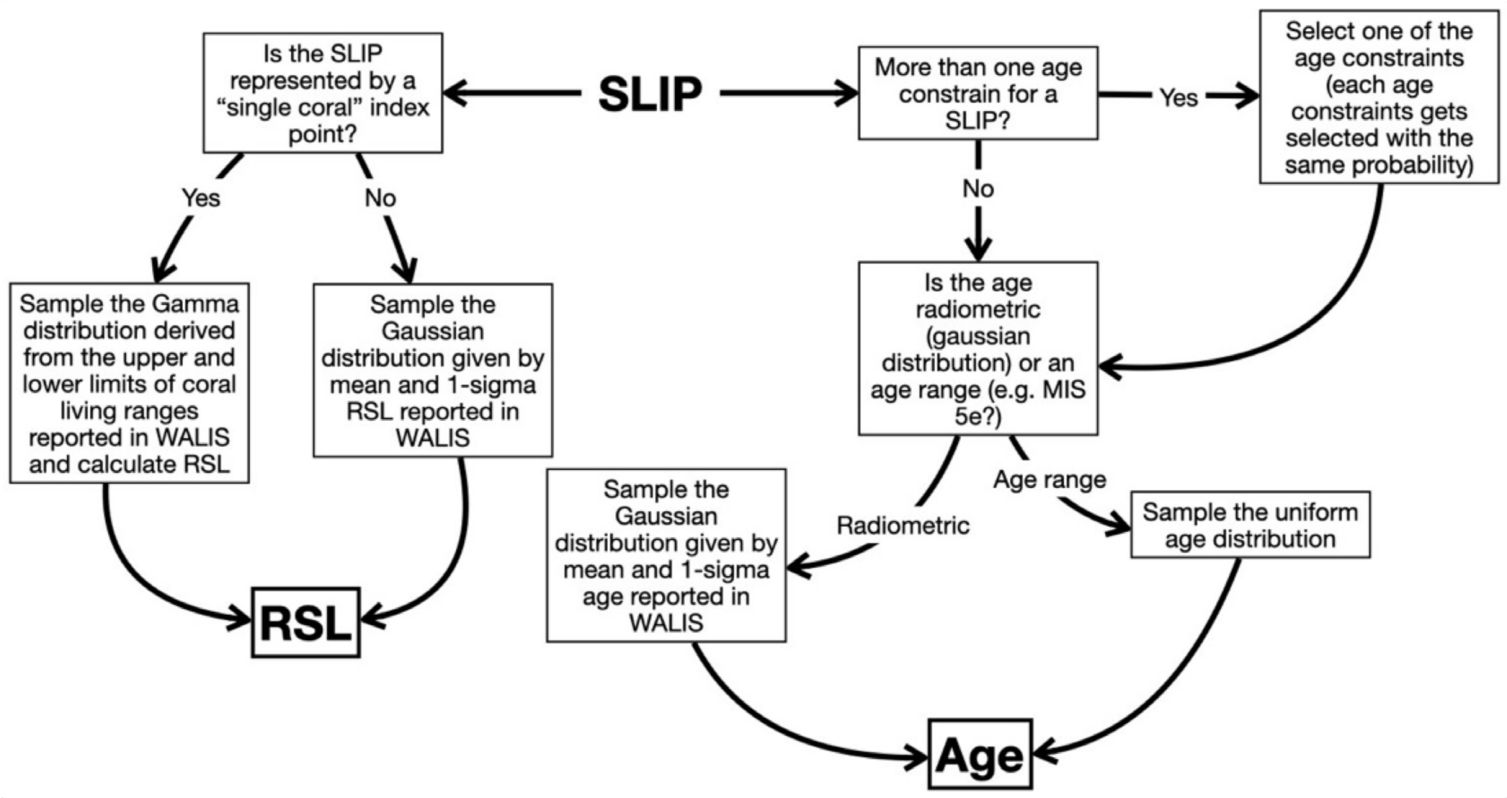
Overview of the processing done on each SLIP within the WALIS Dashboard at each run of the Monte Carlo processing.

The merging method follows the methodology proposed by Garzón (2022) ^9^, which was adapted from the work of Bender *et al.* (2020) ^18^ (code available in Rovere *et al.*, 2020 ^19^). The method consists of a Monte Carlo sampling of RSL and Age for each SLIP within their probabilistic distributions ( Figure 2). In the WALIS Dashboard, the end-user can select how many times per SLIP the workflow shown in Figure 2 is repeated. The WALIS Dashboard limits the number of points per SLIP depending on the number of SLIPs selected for the analysis. Analysis including a large number of samples can be performed by downloading a docker container from the “Download” menu after merging a selection of SLIPs.

After the SLIPs are merged, the user can explore the results in a RSL vs Age plot. The results can then be exported using two different strategies: " *Point cloud download*" or " *Docker container*". The first option exports the resulting point cloud into a CSV file accompanied by a geoJSON file with information about the filters used. The second option creates a docker image accompanied by the required data and code to fully reproduce the results of the data merging. In this way, the results obtained remotely in the Dashboard are fully reproducible on a local machine.

### Operation

***System requirements***

The WALIS dashboard is available both online and offline. The online version only requires a stable internet connection to access the interactive web app. To guarantee the long-term availability of the application, here we explain two alternatives to deploy the application on a local machine.

***Local shiny application***

Users can deploy a local implementation of the shiny application using the {renv} package. This option provides the required files and R packages information. An R installation is a prerequisite for this option.

- 1. Download the WALIS dashboard from GitHub - see *Software availability*
- 2. Open R and install the *renv* package.
  - install.package("renv")
- 3. Open the R Project file (WALIS_Visualization.Rproj). This file should start the process of restoring the dependencies of the project using the Lockfile (renv.lock). You can manually restore the dependencies using the function restore() from the R package *renv*
  - library("renv")
  - restore(renv.lock)
- 4. Open the app.R file.
- 5. Run the App using the runApp() function from the Shiny R package.
  - library("shiny")
  - runApp(your_path/to/app.R)

***Docker image***

A Docker image to run the application is available as part of the application. This docker image allows the application to be fully reproducible as the instructions and computational requirements (e.g., operating system, R packages) to deploy the application inside a software container are automated. The only prerequisite is to have Docker () installed and running on the local machine.

***Download and start-up instructions - Docker***

1. Download the WALIS dashboard from GitHub - see *Software availability*
2. Open Docker to run in the background
3. Open a terminal and access the folder with the application
  - cd WALIS_Visualization
4. Create a Docker container using the Dockerfile image. This process could take hours the first time as it requires setting up all computational requirements.
  - docker build -t ’walis-shiny’.
5. Run the Docker container
  - docker run -p 3838:3838 ’walis-shiny’
6. Open the application in a web browser at *http://localhost:3838*

## Use case

In the following sections, we use the WALIS Dashboard to test a simple hypothesis using RSL data included in WALIS. We want to test the hypothesis that across the island of Curaçao there is a long-term North-South tectonic tilting, similar to the one already suggested for the nearby island of Bonaire ^20, 21^. If present, such tilting should be evident from the paleo RSL record on Curaçao (that is, the local sea-level indicated by SLIPs across the island, uncorrected for any post-depositional displacement).

### Step 1 – Data query

**Figure 3.**
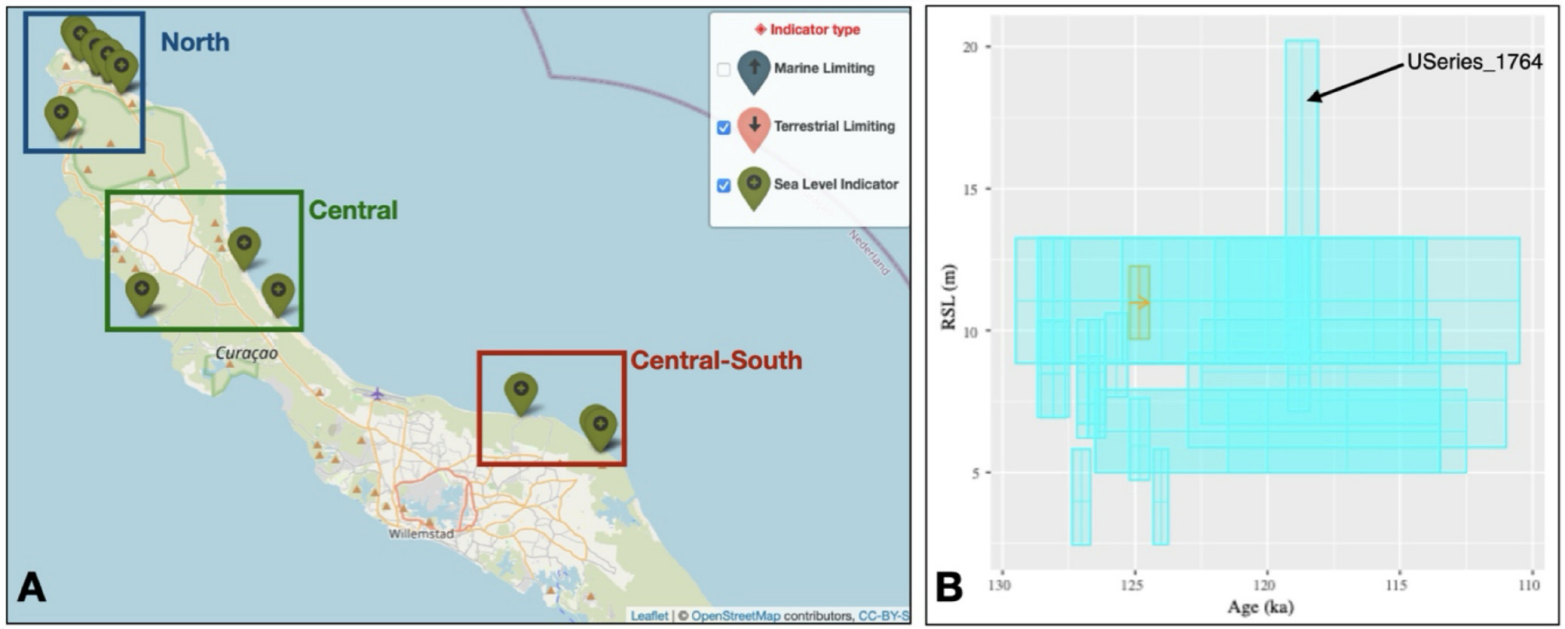
**A**) Map and **B**) RSL vs Age plot from the WALIS Dashboard for the island of Curaçao after the filtering described in the text. The datapoint labelled in **B**) has been excluded before merging the SLIPs, as described in the text.

The first step is to verify that data is present for the island of Curaçao, in the Lesser Antilles. This is done by panning and zooming on the map to the island. From the WALIS Dashboard, we can verify that there is data across large parts of the island, mostly on its Northern, Central, and Central-Southern parts ( Figure 3A). The data spans a large age range (~80-160 ka) and indicate that RSL could have attained elevations between 0 and 20 meters above present ( Figure 3B). For our work, we want to limit the age of the SLIPs to Marine Isotope Stage 5e, i.e., 110-130 ka), and we want to discard marine limiting points (i.e., points that indicates that sea-level was above the measured feature, but for which no relation with the former sea-level is provided). We therefore filter our data using the " *Age filter*" menu to keep only the desired age range, and we turn off from the legend in the main map the "Marine Limiting" points.

### Step 2 – Database Exploration

From the " *Summary Table*" page, we can explore the data and see what literature sources are present. The data in this area has been inserted in the WALIS database by two studies, Chutcharavan and Dutton (2021) ^22^ and Rubio-Sandoval *et al.* (2021) ^23^, who reviewed the works of Schellmann *et al.* (2004) ^24^ and Muhs *et al.* (2012) ^25^. The full references to these studies are present in the full version of WALIS ^8^. In total, in Curaçao, there are 26 SLIPs with ages between 130 and 110 ka, most of them concentrated in the North (n=18), the others in the Central (n=4) and Central-South (n=4) sub-areas ( Figure 3A).

### Step 3 – Merge SLIPs

We use the geographic filter option to select and export (via the " *Summary Table*" page) one CSV file for each sub-area shown in Figure 3A. For each sub-area, we then use the " *Merge SLIP*" function to create a point cloud describing the probability distribution of RSL and age in each sub-area. We use a sample of 10.000 points per SLIP in each area. For each sub-area, we download both the point cloud and the docker container. In the Central-South sub-area, we exclude from merging the index point labelled as "USeries_1764" (labelled in Figure 3B), which is characterized by very large error bars, and therefore may be unreliable.

**Figure 4.**
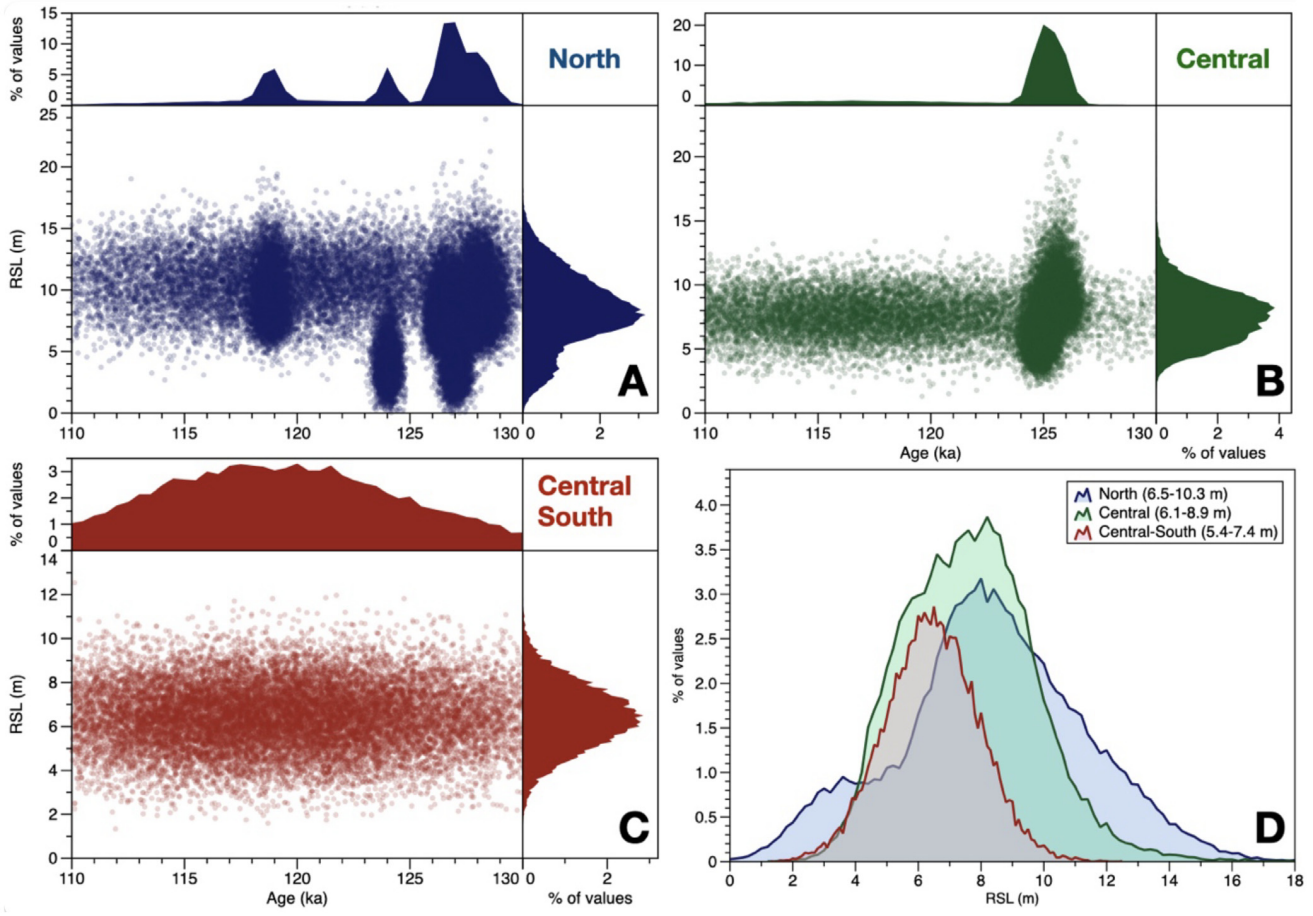
**A**- **C**) Plots showing the point clouds expoted from the WALIS Dashboard after the " *Merge SLIP*" processing. The upper and right panels show the histograms of, respectively, age and RSL derived from the point clouds. **D**) Histograms of RSL for each of the three areas. Within the legend, in parenthesis, are indicated the first and third quartile of each histogram.

We then use the exported point cloud within a graphic software to explore in detail the trends in the data ( Figure 4A-C). From the histograms of RSL in each area ( Figure 4D), we verify that there is an overlap between the RSL records in the North, Central, and Central-South areas. However, the first and third quantiles of the distribution show that, in the North, RSL is slightly higher (6.5-10.3 m) than in the Central (6.1-8.9 m) and Central-South (5.4-7.4 m) parts.

Therefore, the WALIS Dashboard allowed us to extract the data to test a simple hypothesis on the North-South tilting of the island of Curaçao. Standing the results, the hypothesis is rejected, as there is no significant difference between the probability distribution of RSL across the island. However, the data also suggests that there might be some differences (as per the quartiles shown in Figure 4D), therefore highlighting the need for more precise *in-situ* surveys across the island to gauge whether these differences might reflect a different post-depositional deformation signal across the island.

## Conclusions

In this article, we presented an open-source online dashboard developed using R packages to explore the WALIS database. This dashboard is an example of how open-source tools can be used to simplify access to database information for research teams with limited software development or use capabilities. The interactive application consists of three tabs that summarise the database information for researchers to provide a user-friendly point of connection to the information. The application includes basic data processing methods that provide meaningful observations for researchers to start analysing the content of the database. Given the application design, end-users of the application should be able to easily explore the WALIS database before engaging in more complex and time-consuming tasks to understand the database structure. To promote further developments and guarantee the long-term and offline maintenance of the application, the software includes reproducibility strategies such as software containers and dependency management strategies. Similarly, the application is licensed under an MIT permissive free software license, to encourage researcher teams to implement similar interactive visualisation approaches for other databases.

## Ethics and consent

Ethical approval and consent were not required.

## Data Availability

Zenodo: WALIS - The World Atlas of Last Interglacial Shorelines (Ver 1.0 final). https://doi.org/10.5281/zenodo.7348242 This project contains the following underlying data: Atlas_Versions/Ver_1/Ver_1_0_post_review/Output/DB_Structure/Summary_full.csv (CSV file containing the WALIS summary table). Data are available under the terms of the Creative Commons Attribution 4.0 International Public License (CC BY 4.0). Software available from: https://warmcoasts.shinyapps.io/WALIS_Visualization/ Source code available from: https://github.com/Alerovere/WALIS_visualization Archived source code at time of publication: https://doi.org/10.5281/zenodo.4943540 Licence: MIT
