# Supplementary material for: WALIS dashboard: An online tool to explore a global paleo sea-level database
Source: Open Res Eur. 2024 Mar 1;3:114. Originally published 2023 Jul 14. [Version 2] doi: 10.12688/openreseurope.16183.2 (PMC11128055; doi:10.12688/openreseurope.16183.2)
Supplement: Peer review history

# Reviewer report: Reviewer response for version 2

**DOI:** 10.21956/openreseurope.18702.r40182

**Published:** 2024-06-20

**Authors:** Selasi Yao Avornyo

**Affiliations:** 1 University of Ghana, Accra, Greater Accra Region, Ghana

The WALIS Dashboard represents a significant advancement in the field of paleo sea-level research. By providing an intuitive, user-friendly interface to a vast database of sea-level indicators from the Last Interglacial, this tool facilitates greater accessibility and usability of data for researchers, students, and the general public. The open-access nature of the dashboard promotes transparency and encourages collaborative research efforts. The Dashboard's intuitive design and persistent filtering feature across all tabs greatly enhance user experience. The ability to use it both online and offline adds valuable flexibility, making it a highly effective tool. This dashboard demonstrates impressive functionality and ease of use, providing a powerful resource for users.

 I have perused the earlier and current versions; the authors have been responsive to peer review comments, as evidenced by the amendments made from version 1 to version 2. However, peruse your document thoroughly. There are a few typographical and grammatical errors that require attention. I have listed a few below:

**INTRODUCTION:**…quantifiable relationship “ **witha**” former sea level”…should be “ **with a**”.

**THE WALIS DASHBOARD:**…which considers the **reviewer’s** comments and proposes further improvements to the interface, should be **reviewers’**.

**Use Case, Step 1 – Data query:**...points that **indicates** that sea-level was above the measured feature…should be “ **indicate**”.

**Figure 4. A-C) **Plots showing the point clouds **expoted** from…should be “ **exported**”.

 Overall, the WALIS Dashboard is a well-conceived and effectively implemented tool that enhances access to and analysis of paleo sea-level data. The manuscript is thorough and presents the development, functionality, and significance of the dashboard. To ensure its continuous usage and improvement, consider the following as well:

1. Implement a feedback mechanism within the dashboard where users can suggest improvements, report issues, and share how they are using the tool. This will help in continuous improvement and ensure the dashboard meets the evolving needs of the research community.
2. While the manuscript includes a general overview of the code and methods, additional details would enhance replicability. Provide more extensive documentation, including code snippets, setup instructions, and a comprehensive user manual.

Is the rationale for developing the new software tool clearly explained?

Yes

Is the description of the software tool technically sound?

Yes

Are sufficient details of the code, methods and analysis (if applicable) provided to allow replication of the software development and its use by others?

Yes

Is sufficient information provided to allow interpretation of the expected output datasets and any results generated using the tool?

Yes

Reviewer Expertise:

Coastal Processes and Remoting Sensing

I confirm that I have read this submission and believe that I have an appropriate level of expertise to confirm that it is of an acceptable scientific standard, however I have significant reservations, as outlined above.

# Reviewer report: Reviewer response for version 2

**DOI:** 10.21956/openreseurope.18702.r40185

**Published:** 2024-05-25

**Authors:** Stephen Chua

**Affiliations:** 1 Earth Observatory of Singapore,, Nanyang Technological University, Singapore, Singapore

Dear authors,

 I have completed a review of your programme and manuscript entitled: ‘ **WALIS dashboard: An online tool to explore a global paleo sea-level database** **’**. This programme provides a powerful yet user-friendly open-access database with a graphic-user interface (GUI) to tap on the thousands of samples and sea-level indicators dated to the Last Interglacial and older. Previous reviewers have already provided excellent feedback and technical suggestions to the manuscript and dashboard which have been well addressed in version 2. I am in good agreement with their points especially the clarification and substantiation of the Merge SLIPs function.

 The intention of the authors for widespread utilisation of this tool as stated in the Plain language summary (i.e., used by researchers, students and citizens) is laudable. I will thus approach this review from a slightly different angle – that of a non-expert end-user.

**Manuscript comments**

 To achieve this goal, this paper could perhaps expound more on key terms and concepts required by the end users to use the tool effectively. For example, there could be a sentence or two explaining the difference between global mean sea level (GMSL) and relative sea-level (RSL). This is fundamental information for non-experts to understand the premise of this tool in para 1 of the introduction.

*Is sufficient information provided to allow interpretation of the expected output datasets and any results generated using the tool?*

 I answered 'partly' because the lone practice 'case study'  is rather advanced and perhaps a simpler one is needed for non-technical users. I understand from other reviews that there used to be more case studies in version 1 which were removed in version 2. Unfortunately the hyperlink to version 1 is no longer working for me and thus I cannot comment on other case studies. From what I infer from reviewer 1’s comments case study #1 seem to be a straightforward first-order collation of regional sea-level data for understanding RSL magnitude and timing at a region of interest. I think such a case study would aid greater adoption and usage amongst your target user groups.

**Dashboard comments**

 For ease of understanding each sea-level data point, I hope that elevation values can also be provided when a sample is clicked. At the moment I can only view ‘elevation error’ when clicking on a sample point in the map view - I had to access the summary table to see both elevation and the elevation error. Also it would be very helpful if that particular data point gets highlighted in the synthesized sea-level plot on the left window when a sample is selected in the map view on the right window. For example, a site near Gaudulupe, South America has 4 samples RSL 3003 – 3006. It would thus be useful to know which sample refers to which SLIP in the sea-level plot especially when we cannot view elevation values.

 I observed a strange occurrence in the summary table tab. When I minimized text size of the summary table the geographic extent in map view likewise got zoomed in and I lost the delineation of study region. I could not recover the original map extent in summary table view and had to redo in the interactive map view.

 My final point has to do with data management - would the database be continually updated as new studies emerge in the future? 

 Overall, an impressive and valuable tool and excellent contribution to the sea-level community and anyone in relevant fields and interest.

Is the rationale for developing the new software tool clearly explained?

Yes

Is the description of the software tool technically sound?

Yes

Are sufficient details of the code, methods and analysis (if applicable) provided to allow replication of the software development and its use by others?

Yes

Is sufficient information provided to allow interpretation of the expected output datasets and any results generated using the tool?

Partly

Reviewer Expertise:

sea level, paleoenvironmental reconstruction, stratigraphy

I confirm that I have read this submission and believe that I have an appropriate level of expertise to confirm that it is of an acceptable scientific standard.

# Reviewer report: Reviewer response for version 2

**DOI:** 10.21956/openreseurope.18702.r38619

**Published:** 2024-04-05

**Authors:** Jennifer S. Walker

**Affiliations:** 1 Rowan University, Glassboro, New Jersey, USA

I appreciate the authors’ efforts in addressing my previous comments and find that the manuscript has been greatly improved through the revision process. I just have a few final comments below.

   In the WALIS dashboard, on the interactive map page, under the RSL indicator filter, under "Select type of RSL indicator", when I click on an indicator, nothing happens. You can't retain any selection of an indicator, select multiple indicators etc. Therefore, this feature needs to be fixed.

   There are still typos/grammatical errors in the text and in the WALIS dashboard, so they should go through a final editing. To name a few examples in the dashboard: 1) on the interactive map page, under RSL indicator filter, under the uncertainty description, it should read "This variable does not **apply**..."; 2) on the summary table page, there should be a period after 'You can modify these filters in the Interactive map tab'; 3) on the merge SLIPs page, under the Current selection description, 'additional' is spelled incorrectly

Is the rationale for developing the new software tool clearly explained?

Yes

Is the description of the software tool technically sound?

Yes

Are sufficient details of the code, methods and analysis (if applicable) provided to allow replication of the software development and its use by others?

Partly

Is sufficient information provided to allow interpretation of the expected output datasets and any results generated using the tool?

Partly

Reviewer Expertise:

Paleo sea level reconstructions/modeling

I confirm that I have read this submission and believe that I have an appropriate level of expertise to confirm that it is of an acceptable scientific standard.

# Reviewer report: Reviewer response for version 1

**DOI:** 10.21956/openreseurope.17470.r34623

**Published:** 2023-09-14

**Authors:** Jennifer S. Walker

**Affiliations:** 1 Rowan University, Glassboro, New Jersey, USA

This manuscript introduces and details the WALIS dashboard, which is a new interface to explore and interact with sea-level data contained in the World Atlas of Last Interglacial Shorelines paleo sea-level database which has been previously published. The dashboard allows users to quickly explore the available data through a map visualization, sea-level plots, and a series of filters such as the type of indicator, age, or uncertainty. The authors provide their motivation for creating the dashboard and describe how to use the various elements of the interface with detailed examples through three case studies.

 An online open-access tool to investigate an extensive standardized sea-level database is very useful within the sea-level community and for anyone interested in simply exploring available sea-level data in an accessible, interactive way. Having such an interface is a great next step forward from the standardized sea-level databases that have been created across different spatial and temporal scales. Overall I would approve this manuscript with reservations considering my comments below.

**Comments**

 In the last paragraph of the introduction, it is not clear what is meant by ‘one-to-one and many-to-many relationships.’ Could the authors please explain this?

 The interactive map and summary table are valuable tools to have and use case #1 clearly describes how to discover sea level indicators in a way that is simple to replicate. However, I am uncertain about the use of the Merge SLIP feature that is described in use case #2. The method is from a masters thesis that does not seem to be available online and is not fully described in this manuscript. I would suggest the methodology be fully presented in this paper to go through formal peer-review or that this feature is removed from the dashboard until the methodology for the Merge SLIP is published elsewhere and could be added to the dashboard at a later date. As written, it is unclear how the Merge SLIP results should be interpreted, it at all, or if this is just another method to simply view a particular dataset. The dashboard is still a valuable tool with the map, summary, and plotting features to access paleo sea-level data. Further, I was not able to replicate use case #2 because the dashboard crashes when I tried to produce the point cloud and density figures and then resets all of the filters.

 Use case #3 describes how to reproduce the results, which is important by itself, but is not really an example of how to use the dashboard, which should require no coding experience as stated in the introduction. I did not try to replicate use case #3 so cannot comment on its reproducibility, but I might suggest moving this information to a supplemental file for those interested where perhaps an example could be included with how users would perform their own individual analyses.

 Because the manuscript is focused on the accessibility and exploration of data, and I am not sure about the suitability or relevance of use cases #2 and #3, one suggestion might be to add a new use case #2 that is focused on the dashboard and its specific features. Use case #1 is about discovering sea level indicators, so perhaps the authors could have a use case that begins with more of an actual research question and explore how a user could use the dashboard to determine where data is available, what relevant information is available in the summary table, and how having access to the dashboard specifically contributes to advancing the paleo sea-level field.

 While the manuscript is generally clearly written, there is some awkward wording throughout the text, including typos/misspelled words in the text and within the figures (ex. ‘analyse’ spelled incorrectly in Figure 1), the capitalization of words or terms from the dashboard is inconsistent (ex. sometimes only first word of a term is capitalized, other times all words are capitalized – see ‘Age filter’ and ‘RSL Indicator filter’ on page 4), and some unnecessary/repetitive wording or descriptions. Overall the text could be cleaner and would benefit from a readthrough to correct these mistakes and inconsistencies.

 The interface itself is straightforward to use, but would benefit from final editing to fix typos and inconsistent capitalization of categories and terms throughout the various menus to improve the clarity of the dashboard.

 Another comment with the dashboard is that if you move to another window or other internet tab for a period of time, the dashboard will often ‘disconnect from the server’, so that when you go back to the dashboard, anything you were previously viewing has been reset and you would have to start over with finding data, setting filters, etc.

**Answers to peer review form questions**

 Is the rationale for developing the new software tool clearly explained?

- Yes, the rationale is clearly explained in the introduction and its benefits to the scientific community are outlined in the intro and reiterated in the conclusions. Having this dashboard greatly simplifies accessing and visualizing available data as is described in the manuscript.

Is the description of the software tool technically sound?

- Yes, the descriptions of the software to use the dashboard and the offline version are clear and detailed. However, I did not access the offline version.

Are sufficient details of the code, methods and analysis (if applicable) provided to allow replication of the software development and its use by others?

- Partly; the code is available on Github and provides opportunities for replication, as well as through the offline version of the software. However, as stated above, I could not fully replicate use case #2 as the online dashboard kept crashing when I tried to produce the point cloud and density figures. Further, the methods for the Merge SLIP feature are not publicly available and should either be incorporated into this manuscript or the Merge SLIP feature should be removed from the dashboard.

Is sufficient information provided to allow interpretation of the expected output datasets and any results generated using the tool?

- Partly; the interactive map and summary table features produce expected results that can be easily interpreted and are the most valuable part of the dashboard. The manuscript does not provide sufficient information to interpret the Merge SLIP feature as the methods are not described fully or available elsewhere. In the current state of the manuscript and dashboard, the Merge SLIP feature could potentially result in misinterpretation of sea-level data from users since the methods and output are not fully explained.

Is the rationale for developing the new software tool clearly explained?

Yes

Is the description of the software tool technically sound?

Yes

Are sufficient details of the code, methods and analysis (if applicable) provided to allow replication of the software development and its use by others?

Partly

Is sufficient information provided to allow interpretation of the expected output datasets and any results generated using the tool?

Partly

Reviewer Expertise:

Paleo sea level reconstructions/modeling

I confirm that I have read this submission and believe that I have an appropriate level of expertise to confirm that it is of an acceptable scientific standard, however I have significant reservations, as outlined above.

# Reviewer report: Reviewer response for version 1

**DOI:** 10.21956/openreseurope.17470.r33941

**Published:** 2023-08-31

**Authors:** Jessica R. Creveling, Schmitty B. Thompson

**Affiliations:** 1 Oregon State University, Corvallis, Oregon, USA

**Summary of the Article’s Contribution to the Scientific Community**

 In the article titled ‘WALIS dashboard: An online tool to explore a global paleo sea-level database’, authors Garzón and Rovere present a dashboard or ‘graphical user interface’ that enables users with little or no coding knowledge to query the World Atlas of Last Interglacial Shorelines (WALIS) (see Rovere *et al.*, *ESSD*, 2023). The authors present the rationale for this dashboard, a text description of the visualization (map view, table view), and describe research analysis capabilities within this dashboard using three case studies.

 The following comments arise from two co-reviewers’ (Creveling, Thompson) exploration of the online version of the WALIS dashboard; neither of us accessed the offline version through the downloads described in the Operation section and, hence, cannot speak to that functionality.

**Creveling’s Overarching Comments**

 I offer high praise of the interactive map and the summary table components of the WALIS Dashboard. The Interactive Map is a tremendous tool to achieve the goal of case study #1, to discover sea level indicators. The Summary table is a complementary companion to the map-view, and this table guides users into the underlying data/the scientific literature. Overall, the map and table are accessible and easy to navigate. While I offer minor critique on how to enhance these features in the dashboard functionality comments below, I feel that these contributions are ready to share with the community.

 My broader criticisms relate to user case studies #2 and #3.

 For case study #2, the user is guided towards creating a point-cloud of relative sea-level change based using the Merge SLIP tab, and from this encouraged to make research-relevant conclusions, such as “around 130 and 125 ka relative sea level in Bonaire and the northern part of Curaçao was around 12 and 5 m, with a decreasing tendency.” First, I have concerns that the MergeSLIP method does not appear to be peer reviewed––the reference for this is a Master’s thesis that was not accessible in my internet search. While I do not assume that the output is inaccurate, I simply note that it is not evaluable with the given information. I have reservations about the precedent of releasing an unevaluated output to a user community that may lack sea level expertise. Second, I ask that the authors provide a specific, nuanced definition of ‘relative sea level’ in the context of the underlying WALIS data. The example sea level conclusion presented by the authors, that relative sea level was around 12 and 5 m, will likely be perceived very differently based upon expertise. I illustrate this with an example from our own WALIS database entries. If one looked at the ‘relative sea level’ at Newport, Oregon, during MIS 5a (~80 ka), one sees a paleo-RSL of 22.11 m ± 23.04 m. Yet this does not mean that sea level fluctuated between -1 and 45 m at Newport, Oregon during MIS 5a––instead, this is a range that reflects the present-day terrace elevation from 22.5 m ± 22.74 m arising from tectonic warping of the marine terrace over space (the source publication reports this terrace between ~0 – 45 m modern elevation). This “relative sea level” takes no account of regional tectonic uplift. (Perhaps we erred in how we have input this data, leading to this confusion? If so, perhaps others have too?) So it’s from this perspective that when I read in Case study #1 that the authors state that “the sea level plot shows that the relative sea level during the Last Interglacial in this area fluctuated between 2.5 and 20 meters.” that I find myself in disagreement. Have the authors defined ‘relative sea level’ as how the height of the ocean (the geoid) rises relative to the land, and included in that definition land motion from tectonic deformation that accrued long after the indicator formed? The authors state in the Introduction paragraph that corrections for land motions are crucial for global mean sea level––yet I contend that they are crucial for local relative sea level too. Thus, defining “relative sea level” with respect to the WALIS input data is crucial to prevent non-experts from misinterpreting or over-interpret the output.

 Case study #3 (Reproducing the results) shares with users how to reproduce the SLIP Merge analysis presented in case study #2, though this appears to require coding skills more advanced than ‘no coding knowledge’. Whether this remains in the manuscript will depend on the justification for the non-peer reviewed SLIPMerge method. Should it stay, I ask the authors to clarify the first paragraph of this subsection (page 8). I was left with ambiguity about whether this code simply reproduces the relative sea level point cloud in Figure 7 (case study #2) or whether it applies a GIA correction to this point cloud. Please clarify.

 In summary I recommend that the article be revised to: (i) offer clear explanation for how to interpret “relative sea level”, the measure in the sea level plot, given the nature of the underlying WALIS data, and (ii) either eliminate the filtering/merging that generates the relative sea level point cloud or greatly expand the methodological description so that this analysis can be thoroughly peer reviewed. For request (ii) my strong preference would be to ask the authors to separate these two components into distinct publications (removing the point cloud from the Dashboard publication), and then to incorporate the relative sea level point cloud (all ‘analysis’ tools) into the Dashboard after peer review.

**Thompson’s Overarching Comments**

 The problem of effectively accessing and utilizing paleo-shoreline data is well known to the community, and the WALIS interface described in this publication is a strong step towards increasing data accessibility. The authors successfully introduce the issue at hand and present the WALIS dashboard as a solution to improve data accessibility.

 My first broad comment is how the manuscript reports the span and utility of the database. The article abstract presents the contents of the database as “indicators formed during the Last Interglacial (125 ka)” while the database itself contains indicators ranging in age from up to ~450 kyr ago to near present day. Though the project presents itself as the “Atlas of Last Interglacial Shorelines”, the data contained in the database which falls outside of strict last interglacial (LIG) bounds is an important resource. Literature introducing the database to the public shouldn’t exclude components which may be important to those working outside of the LIG. A brief discussion of the scope of data included which falls outside of the LIG will help researchers interested in a wider temporal span utilize the database. Additionally, it is worth mentioning in the introduction that much of the shoreline data is applicable to fields outside of sea level research, such as active tectonics and archeology. Acknowledging a wider range of fields when introducing the database will help broaden the audience for this manuscript (and the database itself).

 I also support the point made above in Creveling’s Overarching Comments that presenting paleo-shoreline elevations as “relative sea level” misses important nuance in interpreting relative sea level data. On many coastlines, especially those near active margins, tectonic movement may lead to significant displacement, sometimes shifting indicators upwards of ~100 m vertically during the interval between the LIG and present day. Interpreting relative sea level from these indicators requires careful correction to remove the tectonic signal, which can significantly obscure the record of sea level change. I appreciate the authors acknowledgement of the role of tectonics and GIA in interpreting global mean sea level from an indicator, but both of these factors also play a large role in interpreting local shoreline data.

 Overall, the WALIS dashboard is a valuable contribution to the sea level community and I appreciate the authors efforts to give an overview in this manuscript. Revisions to address the scope of the data presented in the manuscript and careful reconsideration of the way case study #2 presents the interpretation of sea level data would improve the manuscript.

**Response to Open Research Europe's guiding review questions**

***Is the rationale for developing the new software tool clearly explained?***

 The authors adequately lay out the importance of an interface for the WALIS database as an easy-to-use tool for researchers interested in utilizing (particularly a portion of) the compiled sea level data. As these reviewers are well familiar with, accessing sea level data through the primary publications can be a frustrating endeavor due to inconsistent reporting and standardization, and through the introduction, dashboard overview, and case study #1, the authors make a strong case for how their interactive interface will benefit the greater sea level community.

***Is the description of the software tool technically sound?***

 The authors provide adequate detail of the technical base for the dashboard, supported by providing the full source code powering the dashboard through GitHub and an archived version on Zenodo. As neither reviewer accessed the offline versions of the database, we are not able to speak to the technical descriptions of the offline versions.

***Are sufficient details of the code, methods and analysis (if applicable) provided to allow replication of the software development and its use by others?***

 The base code powering the dashboard itself, being accessible through GitHub and an archived version on Zenodo provides the basis for replicability. The manuscript provides instructions for replicating the full software offline through R and Docker, though neither of us tested these cases. Case study #1 is mostly replicable in the online version, other than some inconsistencies in the parameters outlined in the text (see Line-Specific Comments below for details). Neither reviewer was able to fully replicate case study #2 as displayed in the manuscript instructions and guidance given in Figure 7. Attempting to complete the “merge” with 10,000 points (or a similarly high number) per SLIP as shown in Figure 7 resulted in the dashboard crashing. Other attempts to perform the analysis successfully ran during the initial “Start merging” but crashed when attempting to display the output as a density plot. Merging with the default 5,000 points per SLIP produced a similar but not identical density figure to that illustrated in Figure 7. For case study #3, it is appreciated that the authors provide a method to download the data and files needed to replicate the analysis.

 The major issues that we see with replicability are that the method underlying the “Merge SLIP” analysis is not based on a peer-reviewed publication and that this thesis is not publicly available. While the results of the dashboard can be replicated using the code provided, there is no way to replicate the analysis independent of the underlying dashboard code. 

***Is sufficient information provided to allow interpretation of the expected output datasets and any results generated using the tool?***

 The dashboard ‘filter’ functionalities correctly filter the WALIS database (tested on our own inputs) and provide accessible summaries of the selected data in csv format (as described in use case #1). We feel that these are the most useful and robust outputs that the dashboard provides.

 Importantly, we have concerns that the manuscript’s suggested interpretation in use case #2 lacks important nuance for some users, including ambiguity about interpreting indicator elevations as “relative sea level”. This may potentially mislead future researchers using the dashboard. Additionally, neither the dashboard nor the manuscript provide sufficient information to interpret the Merge SLIP point and density clouds. The lack of accessible, peer-reviewed literature supporting the Merge SLIP analysis prevents robust interpretation of the dashboard output, since the analysis itself has not been validated by the greater community and the literature needed for an individual researcher to closely examine the method is not publicly available.

**Dashboard Functionality Comments**

 The aspect ratio of the world map on the ‘Interactive map’ tab does not allow the user to visualize the entire globe (viewed on both an iMac and laptop running Ubuntu), yet this is never specified. Both reviewers confused the ‘extent:map’ specification as meaning the entire globe. (This becomes clear to a user in the ‘geographic extent’ thumbnail in the Summary table tab. For example, at the maximum zoom out, to include all of North America in the default map almost none of South America can be included in the map). This matters because the corresponding ‘sea level plot’ then has an unspecified geographic filter. When we drag the map around––at the same zoom and with the filter saying ‘extent:map’ throughout––the ‘sea level plot’ updates with the map area, but at no point can we see the ‘sea level plot’ for the entire globe. It appears that a user can never view a sea level plot for the entire globe. We request that the default “Interactive map” be the full globe. We request that the ‘extent:map’ clarify whether the whole globe, or a subset of the globe is selected, and that any further sub-selection of the map area (by manual polygon versus zoom-in area)  have a more specific filter label.    

 We would appreciate a ‘Reset’ button to return all filters to default ranges.

 For Summary Table, can you clarify the meaning of ‘blank’ entries for the user? This may be clear to those who have uploaded data, but perhaps not to those who are coming with fresh eyes.

 For the filter such as age range and elevation error, it would be beneficial to add an option to manually enter in the desired numeric range. While the sliders work well in some scenarios, for ranges spanning large numeric intervals (such as those in the age range), selecting specific values solely with the slider may be difficult or inaccessible to some users.

 The dashboard crashes at times when developing a density cloud, particularly when high Point per SLIP values are used in the merging options.

**Line-specific Comments**

- Would benefit from copy-editing; grammatical errors introduce some ambiguity about the dashboard’s function.
- Please clarify what you mean by ‘database consists of multiple tables with one-to-one or many-to-many relationships’ (page 3).
- Please clarify what you mean in the sentence ‘In our design … as proposed by 4’.
- The text states that the map visualization displays sea level indicators with pop-ups with additional metadata on elevation measurements and dating, etc.. (page 4), yet we find that this metadata only includes the indicator code (RSL_####) with no additional data as described. Please update the pop-up or this text accordingly.
- The authors state that the platform includes notes explaining the temporal and relative sea-level dimensions for each of the nine types of sea level indicators (page 5), though we could only find information on the temporal aspects when hovering our cursor over the nine symbols. Information about the sea-level meaning is missing.~
- The ‘Merge SLIP’ tab title (page 5) is jargon, please consider a phrase more understandable to the non-sea–level expert end user.
- Change 130.00 to 135.00 in restriction #2 of the numbered list in user case #1 (page 7).
- One page 7, the text for use case #1  reads “(e.g., 135 to 110 ka to include additional data)” when describing the age filter menu - please clarify whether this is a theoretical example or a part of the use case, as in order to replicate the sea level plot in figure 6, the age range had to be 135 - 110 kyr (as listed in the example filter diagram in figure 6), not the 130 - 115 kyr listed in item 2 of the restrictions for use case #2.
- For Figure 6a, you only specify the RSL filters ‘elevation error’ but not the ‘percentile’ and ‘uncertainty’. Please specify the former to make this case study more easy to replicate.
- Please clarify what you mean by ‘relative sea-level indicators can have more than one constraint’ (page 8).
- Please clarify what you mean by ‘Uniform distribution’ in ‘For example, sea level indicators in which the age calculation comes from a Uniform distribution from a Marine Isotope Stage (MIS) assignment’ (page 8).
- Please clarify what you mean by ‘remove individual sea-level indicators by code’ (page 8).
- In Figure 1, please restructure elements of the figure to clarify the relationship between the WALIS database itself, the archived copy on Zenodo, the contributors, and the end users.
- We could not reproduce Figure 7 from the information in the text/figure. Please offer more information to replicate this case study.

Is the rationale for developing the new software tool clearly explained?

Yes

Is the description of the software tool technically sound?

Yes

Are sufficient details of the code, methods and analysis (if applicable) provided to allow replication of the software development and its use by others?

Partly

Is sufficient information provided to allow interpretation of the expected output datasets and any results generated using the tool?

Partly

Reviewer Expertise:

Stratigraphy, sea level.

We confirm that we have read this submission and believe that we have an appropriate level of expertise to confirm that it is of an acceptable scientific standard, however we have significant reservations, as outlined above.
